# Optimising AVATAR therapy for people who hear distressing voices: study protocol for the AVATAR2 multi-centre randomised controlled trial

**DOI:** 10.1186/s13063-021-05301-w

**Published:** 2021-05-25

**Authors:** Philippa Garety, Clementine J. Edwards, Thomas Ward, Richard Emsley, Mark Huckvale, Paul McCrone, Mar Rus-Calafell, Miriam Fornells-Ambrojo, Andrew Gumley, Gillian Haddock, Sandra Bucci, Hamish McLeod, Amy Hardy, Emmanuelle Peters, Inez Myin-Germeys, Thomas Craig

**Affiliations:** 1 Institute of Psychiatry, Psychology & Neuroscience, King’s College London, London, UK; 2 South London & Maudsley NHS Foundation Trust, London, UK; 3 University College London, London, UK; 4 University of Greenwich, London, UK; 5 Mental Health Research and Treatment Center, Faculty of Psychology, Ruhr-Universität Bochum, Bochum, Germany; 6 South London & Maudsley NHS Foundation Trust, London, UK; 7 University of Glasgow, Glasgow, UK; 8 NHS Greater Glasgow & Clyde, Glasgow, UK; 9 University of Manchester and the Manchester Academic Health Sciences Centre, Manchester, UK; 10 Greater Manchester Mental Health NHS Foundation Trust and the Manchester Academic Health Sciences Centre, Manchester, UK; 11 KU Leuven, Leuven, Belgium

**Keywords:** Auditory hallucinations, Psychosis, Psychological intervention, Digital health technology, Randomised controlled trial

## Abstract

**Background:**

AVATAR therapy is a novel intervention targeting distressing auditory verbal hallucinations (henceforth ‘voices’). A digital simulation (avatar) of the voice is created and used in a three-way dialogue between participant, avatar and therapist. To date, therapy has been delivered over 6 sessions, comprising an initial phase, focusing on standing up to a hostile avatar, and a second phase in which the avatar concedes and focus shifts to individualised treatment targets, including beliefs about voices. The first fully powered randomised trial found AVATAR therapy resulted in a rapid and substantial fall in voice frequency and associated distress that was superior to supportive counselling at 12 weeks. The main objective of this AVATAR2 trial is to test the efficacy of two forms of AVATAR therapy in reducing voice-related distress: AVATAR-brief (standardised focus on exposure, assertiveness and self-esteem) and AVATAR-extended (phase 1 mirroring AVATAR-brief augmented by a formulation-driven phase 2). Secondary objectives include the examination of additional voice, wellbeing and mood outcomes, the exploration of mediators and moderators of therapy response, and examining cost-effectiveness of both forms of therapy compared with usual treatment (TAU).

**Methods:**

This multi-site parallel group randomised controlled trial will independently randomise 345 individuals to receive AVATAR-brief (6 sessions) plus TAU or AVATAR-extended (12 sessions) plus TAU or TAU alone (1:1:1 allocation). Participants will be people with a diagnosis of schizophrenia spectrum and other psychotic disorders who have heard distressing voices for more than 6 months. The primary outcome is the PSYRATS Auditory Hallucinations Distress dimension score at 16 and 28 weeks, conducted by blinded assessors. Statistical analysis will follow the intention-to-treat principle and data will be analysed using linear mixed models. Mediation and moderation analyses using contemporary causal inference methods will be conducted as secondary analyses. Service costs will be calculated, and cost-effectiveness assessed in terms of quality-adjusted life years accrued.

**Discussion:**

This study will clarify optimal therapy delivery, test efficacy in a multi-site study and enable the testing of the AVATAR software platform, therapy training and provision in NHS settings.

**Trial registration:**

ISRCTN registry ISRCTN55682735. Registered on 22 January 2020. The trial is funded by the Wellcome Trust (WT).

## Administrative information

The order of the items has been modified to group similar items (see http://www.equator-network.org/reporting-guidelines/spirit-2013-statement-defining-standard-protocol-items-for-clinical-trials/).

**Table:** 

Title {1}	Optimising AVATAR therapy for distressing voices: the AVATAR2 multi-centre randomised controlled trial.
Trial registration {2a and 2b}.	ISRTCN registry, reference: [ISRCTN55682735] on 22/01/20.
Protocol version {3}	1.2
Funding {4}	An Innovations Project Award from the Wellcome Trust.
Author details {5a}	Institute of Psychiatry, Psychology & Neuroscience, King’s College London. South London & Maudsley NHS Foundation Trust NIHR Biomedical Research Centre CJE, PAG, TW, TC, EP, AH. RE Mental Health Research and Treatment Center Faculty of Psychology Ruhr-Universität Bochum MRC University of Manchester, GH, SB University of Greenwich PM KU Leuven IMG University College London MFA, MH Institute of Health and Wellbeing, University of Glasgow HM, AG
Name and contact information for the trial sponsor {5b}	Co-Sponsor: King’s College London Professor Reza Razavi, Room 5.31, James Clerk Maxwell Building, 57 Waterloo Road, London, SE1 8WA. 02078483224 reza.razavi@kcl.ac.uk Co-Sponsor: South London & Maudsley NHS Trust R&D Department, Room W1.08 Institute of Psychiatry, Psychology & Neuroscience (IoPPN) De Crespigny Park, London, SE5 8AF. 02078480339 slam-ioppn.research@kcl.ac.uk
Role of sponsor {5c}	The sponsor and funders will monitor the governance of the study and milestones for completion and will have no other role in the design, collection, analysis or interpretation of data or the decision to submit to report for publication.

## Introduction

### Background and rationale {6a}

Voices are the most commonly reported form of auditory hallucinations [1] heard by as many as 70% of people diagnosed with schizophrenia-spectrum disorders [2]. They are often associated with high levels of distress and can have a profound impact on everyday life, with typical content involving threats, commands, and abusive comments. Voices frequently persist for many years despite optimal pharmacotherapy, and the currently recommended psychological therapy for voices, cognitive behavioural therapy for psychosis (CBTp) is effective [3] but only for just over 50% of people [4]. CBTp also involves delivering a relatively lengthy treatment, which presents significant barriers to access [5]. Consequently, there is considerable interest in the development of novel therapies that are informed by CBTp principles, but which are both shorter and capable of being delivered by a wider workforce.

Typically, voices have ascribed ‘characterful’ identities and are experienced as profoundly ‘real’ [6] with three quarters of people who hear voices reporting an associated mental image [7]. Cognitive models of voices highlight the role of beliefs about voices in voice-related distress (notably relating to omnipotence and malevolence) [8]. Social rank theory [9] has been applied to voices with evidence that the inferiority and powerlessness people commonly experience in relation to their voices may mirror key social relationships, which for some include experiences of trauma [10]. This relational perspective informs psychological approaches to distressing voices that focus on the interpersonal relationship between the voice-hearer and the voice [11] and seek to understand the voice in the broader context of the person’s life [12, 13].

AVATAR therapy is one such relational approach. A digital representation (avatar) of the person’s main distressing voice is created, informed by a detailed assessment of the voice (including verbatim content, voice characterisation, the nature of voice-hearer relationship and developmental history). Bespoke software transforms the voice of the therapist to match the auditory characteristics of the voice. The person then creates a visual representation of the voice in line with the ascribed identity and character. The two processes are combined to produce the avatar, through which the therapist can interact with the voice-hearer using a console to switch between speaking in the transformed avatar voice and their own voice. AVATAR therapy as delivered to date comprises two phases. In the first phase, (Exposure and Assertiveness) the avatar delivers verbatim voice content (including threats and abuse) and the person practices assertive responding. Over time the avatar becomes less hostile as the person develops increased power and control within the dialogue. This cues a second phase with formulation-driven therapeutic targets, which can include work on beliefs about voices, self-concept and trauma [14].

Following a proof-of-concept study [15], we have conducted a fully powered pragmatic, single blind single-centre RCT [16] comparing AVATAR therapy with supportive counselling (SC) delivered over 7 weekly sessions. This showed that AVATAR therapy resulted in a rapid and substantial reduction in the frequency and distress (PSYRATS-AH) and power of voices (BAVQ-R), that was significantly superior to SC at 12 weeks (the primary outcome). The observed differences at 12 weeks were larger than anticipated with a between-group effect size of 0.8 suggesting that it is a more effective therapy for voices than the alternatives currently available. At 24 weeks, the differences between the two arms were no longer statistically significant, although the clinically significant improvements in the AVATAR therapy scores on primary outcome were sustained.

The lack of a treatment as usual (TAU) arm in this study made the absence of difference between the two active treatment groups at 24 weeks hard to interpret. Similarly, attempts to understand the mechanisms of action for AVATAR therapy are more challenging in the context of an active treatment control. With respect to possible factors which may influence treatment response, a recent analysis of in-session ratings (collected only in the AVATAR therapy arm) explored self-reported anxiety and the sense of voice presence (i.e. the extent to which speaking to the avatar was experienced as like talking to the everyday voice). Whilst sense of voice presence remained consistently high across all sessions, there was a significant reduction in anxiety which was driven by a rapid change during sessions 1–3 (phase 1). Notably, improvements in voice frequency and overall severity at follow-up were predicted by an interaction between overall anxiety reduction and sense of voice presence [17]. Allied to clinical reports of significant early improvements [14], this raises the question as to whether a consistent phase 1 focus on exposure and assertiveness to a valid representation of the voice might be sufficient for some individuals; for example, where distress is closely linked to fear, anxiety and relational submissiveness. There would be fewer barriers to training and dissemination of a standardised phase one-based AVATAR therapy compared to the extended therapy, potentially facilitating implementation both across the UK and in other settings worldwide where therapists trained in formulation-based psychological therapies are in short supply.

On the other hand, phase 2 of AVATAR therapy targets developmental and maintenance (‘here and now’) processes specific to an individual formulation. The phase 1 focus on anxiety reduction and assertiveness is extended in a way that may be necessary for optimally effective lasting change where distress is associated with other cognitive, emotional and relational processes. Notably, phase 2 targets beliefs about voices (relating to perceived power and malevolent intent) and aims to facilitate a change in the relationship with the voice which is grounded in autobiographical context and personal meaning, e.g. where voices reflect past experiences of disempowerment and social defeat, the dialogues target emotional resolution and updated views of the self. We will examine these key treatment targets (anxiety and beliefs about voice power and malevolence) as mediators of treatment effects.

The more complex and personalised phase 2 dialogue may be particularly relevant for voices that are experienced as characterful social agents [18–20], i.e. voices with ascribed attitudes, intentionality, and personality (their own ‘character’). Ward and colleagues [21] found that one third of individuals in their AVATAR therapy sample reported a highly characterised dominant voice (e.g. voices associated with a family member or identifiable ‘stranger’ with a consistent background and personality). Specifically, these individuals showed increased interaction (time in conversation) with the avatar compared to those with non-complex characterisation (voices with no clear identity or with only ‘one-dimensional’ character, e.g. ‘an angry, punishing spirit’). Whilst this suggests that the degree of voice characterisation can affect engagement in active dialogue, it remains an open question as to whether it might influence treatment outcome. We plan to address this question in the current trial design, randomising to the two forms of therapy plus TAU, AVATAR-Brief (Phase 1 only) or AVATAR-Extended (phase 1 plus phase 2), or to TAU alone, stratifying by baseline level of characterisation. We hypothesise that whether the voice hearer’s main voice is more or less highly characterised influences (moderates) the treatment effect of level of therapy compared to TAU. If so, this would provide important information concerning targeted treatment. Additional exploratory moderation analyses will consider other demographic and clinical factors which might plausibly influence treatment effects. These include attachment and trauma history, given the relational nature of the AVATAR approach, as well as clinical characteristics found to affect engagement in psychological interventions (e.g. negative symptoms [22] and post-traumatic stress disorder (PTSD) symptomatology [23–25]). Our previous work investigated the cost-effectiveness of AVATAR therapy compared to supportive counselling and found favourable results (paper in preparation). This study will enable us to make a more robust estimate of cost-effectiveness.

### Objectives {7}

The trial will address questions of treatment efficacy and cost-effectiveness of brief and extended versions of therapy and their implementation in NHS settings, including testing the provision of the integrated and enhanced software platform for the delivery of therapy employed in the clinical trial, together with the operational and therapy manuals.

#### Hypotheses

1. AVATAR-brief will be more effective in reducing voice-related distress, total voice severity and voice frequency than TAU at post-treatment (16 weeks) and follow up (28 weeks)
2. AVATAR-extended will be more effective in reducing voice-related distress, total voice severity and voice frequency than TAU, at post-treatment (16 weeks) and follow-up (28 weeks)
3. AVATAR-extended will reduce perceived omnipotence and malevolence (BAVQ-R) compared to TAU and these improvements will mediate change in the primary outcome.
4. In both AVATAR-brief and AVATAR-extended treatment effects on the primary outcome will be mediated by anxiety reduction, as measured by Experience Sampling Methodology (ESM) in daily life.
5. Greater baseline complexity of voice characterisation will moderate the treatment effects of AVATAR-brief and AVATAR-extended compared to TAU. Other clinical characteristics will be explored as potential moderators.
6. AVATAR-brief and AVATAR-extended will both have favourable incremental cost-effectiveness ratios compared to routine care.

### Trial design {8}

A three-arm parallel-group superiority randomised controlled trial, with 1:1:1 allocation and blinded assessors, to test the efficacy of AVATAR therapy (Brief and Extended forms) in people diagnosed with schizophrenia spectrum disorders in reducing voice-related distress when added to treatment as usual (TAU) compared to TAU alone.

## Methods: participants, interventions and outcomes

### Study setting {9}

Four UK research sites will take part in the trial: Institute of Psychiatry, Psychology & Neuroscience (KCL), University of Manchester, University of Glasgow and University College London. At least two local NHS sites will be attached to each of these research sites and therapy will take place in community settings.

### COVID-19 adaptations to the protocol

In response to the global pandemic, the research team have made adaptations to the protocol to enable AVATAR2 to continue in the event of social distancing restrictions being in place. These adaptations to the protocol are detailed in the recruitment, assessment and therapy delivery sections below. Where possible, the AVATAR2 team will see participants face-to-face as detailed in this protocol; the changes outlined will be applied when this is required by NHS trust and University guidance.

### Eligibility criteria {10}

The inclusion criteria for the study are as follows: (1) aged 18 years or over; (2) currently under the care of a specialist mental health team (inpatient and outpatient settings); (3) have current frequent and distressing voices, (as measured by a score of at least 1 on each of the intensity of distress and frequency items of the PSYRATS (Voices) scale), persisting for at least 6 months and spoken in English; (4) speak and read English to a sufficient level to provide consent and complete the assessment procedures; and (5) a clinical diagnosis of Schizophrenia spectrum disorder (ICD10 F20–29) or affective disorder with psychotic symptoms (ICD-10 F30–39, subcategories with psychotic symptoms) as determined through clinical records and additional consultation with the clinical team if unclear. Criteria for exclusion are as follows: (1) primary diagnosis of substance disorder, personality disorder or learning disability; (2) lacking capacity to consent; (3) profound visual/hearing impairment or insufficient comprehension of English to be able to engage in assessment or therapy; (4) currently undertaking individual psychological therapy for voices; and (5) currently experiencing an acute mental health crisis.

Therapy will be delivered by mental health clinicians, mostly psychologists, with experience of delivering psychological therapy to people with psychosis.

### Who will take informed consent? {26a}

Potential participants will initially be contacted by a member of their clinical team in the participating NHS sites, who believes them to be eligible, inviting them to learn more about the study. They may also be contacted if they have indicated they are interested in research through their NHS Trust (e.g. “Consent for Contact”) and can also self-refer directly into the study. Once they have expressed their interest or agreed to be contacted, they will be approached by a research assistant who will confirm eligibility criteria and obtain consent to participate in the research.

#### COVID-19

The information sheet and consent form detail the changes that will be made to the protocol during social distancing.

We will continue recruitment of participants as described above where possible during social restrictions using remote processes. The research team will maintain communication with clinical teams remotely and referrals will be made in the same manner. If face-to-face contact is not possible, alternative consent options will be utilised as follows:

1. Obtaining consent using an electronic signature or online consent form;
2. Recording verbal consent via video calling or telephone. These recordings will be stored securely and linked to the participant code, on university computers, separate from any identifying details.

### Additional consent provisions for collection and use of participant data and biological specimens {26b}

On the consent form, participants will be asked if they agree to the use of their data should they choose to withdraw from the trial. Participants will also be asked for permission for the research team to share relevant data with people from the Universities taking part in the research or from regulatory authorities, where relevant. This trial does not involve collecting biological specimens for storage.

## Interventions

### Explanation for the choice of comparators {6b}

The previous trial of AVATAR therapy showed it was effective post-treatment at 12 weeks, compared with an active control treatment (supportive counselling) but the lack of a TAU only arm meant that it was difficult to interpret the absence of a difference between the two active treatment groups at 24 weeks. The next stage of treatment evaluation and research therefore calls for a TAU control comparator before wider dissemination.

Additionally, there are questions about the treatment. As described above, AVATAR therapy as delivered in the previous trial comprises two phases. The trial therapists noted that phase 1 in itself was a powerful intervention for a sizeable number of people, which was supported by evidence within the AVATAR therapy arm of significant anxiety reduction during sessions 1–3 [17]. On the other hand, for some people, anxiety reduction alone may be insufficient to produce optimally effective lasting change, and therefore an approach including both phases may be necessary (see the ‘Introduction’ section). This trial will therefore include two treatment arms, AVATAR-brief (6 sessions) and AVATAR-extended (12 sessions), each combined with TAU, to allow an evaluation and comparison of both these approaches with TAU alone, with an additional health economics evaluation of the incremental cost-effectiveness ratios of each approach compared with TAU.

### Intervention description {11a}

AVATAR-brief consists of six individual, face-to-face sessions (in addition to an initial clinical assessment session including avatar creation), delivered by trained therapists (with previous experience working in psychosis), assisted by the avatar software described previously. Therapy focuses on the exposure/assertiveness component and will follow detailed session-by-session guidance in a written AVATAR clinical manual. At the end of each interaction with the avatar, the therapist will return to the room and support the participant to answer questions regarding their experience of the interaction (perceived hostility, anxiety, sense of voice presence) presented either on the screen or in a booklet (at therapist’s discretion).

AVATAR-extended consists of twelve individual, face-to-face sessions (in addition to the initial assessment and avatar creation session), delivered by trained therapists, assisted by the AVATAR therapy software described previously. It includes both phases of treatment as described earlier and detailed in the AVATAR clinical manual. The participants will also complete the same questions at the end of each interaction with the avatar as described above. In line with the clinical manual, the number of sessions for both AVATAR-brief or AVATAR-extended is permitted to vary by no more than two sessions added or removed; any such change will be guided by the clinical judgement of the therapist and in collaboration with the participant.

It is necessary to have access to two private rooms to conduct the therapy; additional equipment includes a laptop (with a webcam and microphone) and secure network, the AVATAR therapy software and the participant must have access to a device on which they can listen to recordings of the sessions (this will be provided by the study team as required). Face-to-face sessions will mostly be conducted at a clinical research facility or local clinical team settings.

Approximately 20 clinicians will be trained to deliver AVATAR therapy during the trial. This will include a core team of 8 trial therapists and clinicians at local NHS sites who will be trained with a view to delivering a smaller number of cases and building therapy and training capacity in the NHS. Training will involve initial orientation to the clinical and technical manuals followed by sessions (face-to-face or remote) focusing on the rationale, therapy targets and delivery of AVATAR-brief and AVATAR-extended. Initial pilot cases will be closely supervised by someone with experience of AVATAR therapy delivery. Training cases will be assessed for treatment fidelity and therapist competence using tools which have been developed for routine monitoring throughout the trial (see the ‘Strategies to improve adherence to interventions {11c}’ section). Therapy supervision during the trial will include individual and group-based supervision delivered from the main site (King’s College London) in addition to individual and peer-based supervision at each local site.

For both AVATAR-extended and AVATAR-brief arms, in-session measures assessing anxiety, perceived hostility, sense of presence and sensed presence will be completed at the end of each interaction with the avatar (items adapted from [26, 27]). The Working Alliance Inventory (WAI) Short-Form will be used to assess the quality of the therapeutic alliance from the perspective of the participant- this will occur at Session 4 (AVATAR-extended and AVATAR-brief) and repeated in Session 10 (AVATAR-extended only) if therapy delivery changes during the course of intervention (remote or face to face). Therapy process activities (e.g. between session phone contact or messaging and clinical team liaison) will be routinely collected to inform future implementation.

The AVATAR Therapy software [28] has been CE marked and registered with the MHRA as a class 1 medical device by Avatar Therapy Ltd. (www.avatartherapy.co.uk).

#### COVID-19

AVATAR therapy, using the same clinical manual, will be delivered remotely using a bespoke, secure, end-to-end encrypted video-call system which will enable the delivery of the therapy in accordance with the clinical manual. Where the participant does not have access to appropriate equipment at home, the therapist will work with the person to find a solution that allows therapy to take place remotely. Where necessary, AVATAR2 will provide the hardware to the participant (e.g. tablet or computer), which would be returned to the research team at the end of the therapy period.

In addition to the protocol as outlined above, there will be one remote therapy set-up session where the therapist will explain how the bespoke AVATAR therapy video call system works and problem solve the set-up of the remote therapy sessions.

### Criteria for discontinuing or modifying allocated interventions {11b}

Apart from the change in number of sessions (plus or minus 2) described above, to take account of clinical circumstances, the intervention may be discontinued if the participant requests this or if the clinician judges, in consultation with the wider supervisory team, that the intervention is associated with a significant worsening of mental health. In this case an Adverse Event form would also be completed. It will be made clear to each participant that, should they find any aspect of the research distressing, including the intervention, and/or no longer wish to continue, they will be able to withdraw without this impacting on their usual clinical care in any way.

### Strategies to improve adherence to interventions {11c}

The number of active sessions attended (i.e. sessions involving active avatar dialogue) by the participant will be measured to assess treatment adherence. Fidelity to the clinical manual will be assessed by the therapist completing a session-by-session checklist of specified components (these will also be used during training and ongoing supervision- see above). Each session will be audio recorded with consent. After completion of training, therapist competence will be assessed by an expert in AVATAR therapy for general/clinical and AVATAR-specific skills using ratings adapted from the first AVATAR therapy trial and allowing for differing skill requirements for each level of therapy. Each trial therapist will be rated for competence based on the review of early, mid and late session therapy delivery for at least one completed intervention. Ratings will be conducted for two cases for therapists who deliver completed therapy with more than five participants.

### Relevant concomitant care permitted or prohibited during the trial {11d}

Participation will not alter clinical treatment decisions about medication and additional psychological and psychosocial interventions which remain the responsibility of the clinical team. The antipsychotic medication prescribed to participants in the study and psychological and psychosocial interventions provided will be recorded.

### Provisions for post-trial care {30}

There is no provision for post-trial care in the study and participants will remain under the care of their usual mental health services. The trial is covered by the Sponsor’s (King’s College London) indemnity insurance.

### Outcomes {12}

The primary outcome for the study is reduction at end of treatment (16 weeks) and follow up (28 weeks) in the distress associated with voices, as measured by the distress dimension of the Psychotic Symptoms Rating Scale (PSYRATS-AH) [29, 30].

We note the recent CONSORT-SPI guidance which recommends minimising the distinction between primary and secondary outcomes, therefore all outcomes will be reported at the end of the trial [31].

All measures are detailed in Table 1.

**Table 1:** AVATAR2 measures

	Measure	Time-point*
Distress, Frequency and Severity of Voices Outcome Measures	Psychotic Symptom Rating Scales – Auditory Hallucinations – a dimensional measure of hallucinations [29]. Includes Distress dimension (primary outcome), Total score (secondary outcome), and Frequency dimension (secondary outcome) [30]. Hallucinations Remission Score	Screening, 1, 2, 3
Other Voices Outcome Measures	Beliefs about Voices Revised (BAVQ-R) [32]. Voices acceptance and action scale (VAAS) [33]. First item (power) from the Voice Power Differential Scale [34].	1, 2, 3
Experience Sampling Methodology	AVATAR2 ESM Questionnaire [35].	1,2,3
	AVATAR2 ESM Debrief Questionnaire [36].	1,2,3
Other Secondary Outcome Measures	Mood: Beck Depression Inventory-II [37].	1, 2, 3
	Anxiety: Depression Anxiety and Stress Scales (DASS-21) [38].	1,2,3
	Quality of Life: Warwick-Edinburgh Mental Well-being Scale [39].	1, 2, 3
	Delusions: Psychotic Symptoms Rating Scale (PSYRATS-DEL) [29].	1, 2, 3
	Service User Led Outcome Measure: Choice of Outcome in CBT for Psychosis (CHOICE) Short Form [40].	1,2,3
	Trauma related symptoms – International Trauma Questionnaire (ITQ) [41].	1, 2
Health Economic Outcome Measures	Service use and costs: costs of the intervention (based on staff time, overheads and equipment) and the costs of other key health and social care services calculated using the [42].	1, 2, 3
	EQ-5D-5L [43] to measure QALYs	1, 2, 3
Baseline Clinical Characteristics	Voice Characterisation Checklist: Assesses the complexity in characterisation of the dominant voice. Rated as more vs. less highly characterised based on presence/absence of ascribed identity (e.g. known, name), physical characteristics (e.g. age, gender, accent) and psychosocial characteristics (e.g. intentions, thoughts, relationships)-see [21]. Including two items from the Voice Phenomenology Interview [44].	1
	Fearful Attachment Style Item from Relationships Questionnaire [45].	1
	Patient perceived trauma-voice links - Trauma Voice Associations Questionnaire (TVAQ)	1
	Trauma history - Mini-Trauma And Life Events (TALE) checklist [46].	1
	Clinical Assessment Interview for Negative Symptoms (CAINS) [47].	1
	Identification of positive symptoms – Initial items from the Schedules for Clinical Assessment in Neuropsychiatry (SCAN) 2.1 [48].	1
	Detailed assessment of positive symptoms - Scale for the Assessment of Positive Symptoms [49].	1
Covid-19 Impact	Covid-19 Context Questionnaire to assess individual circumstances during pandemic and change in voice experiences.	1, 2, 3

^a^1 = baseline, 2 = 16 weeks post-randomisation, 3 = 28 weeks post-randomisation

### Participant timeline {13}

**Fig. 1:**
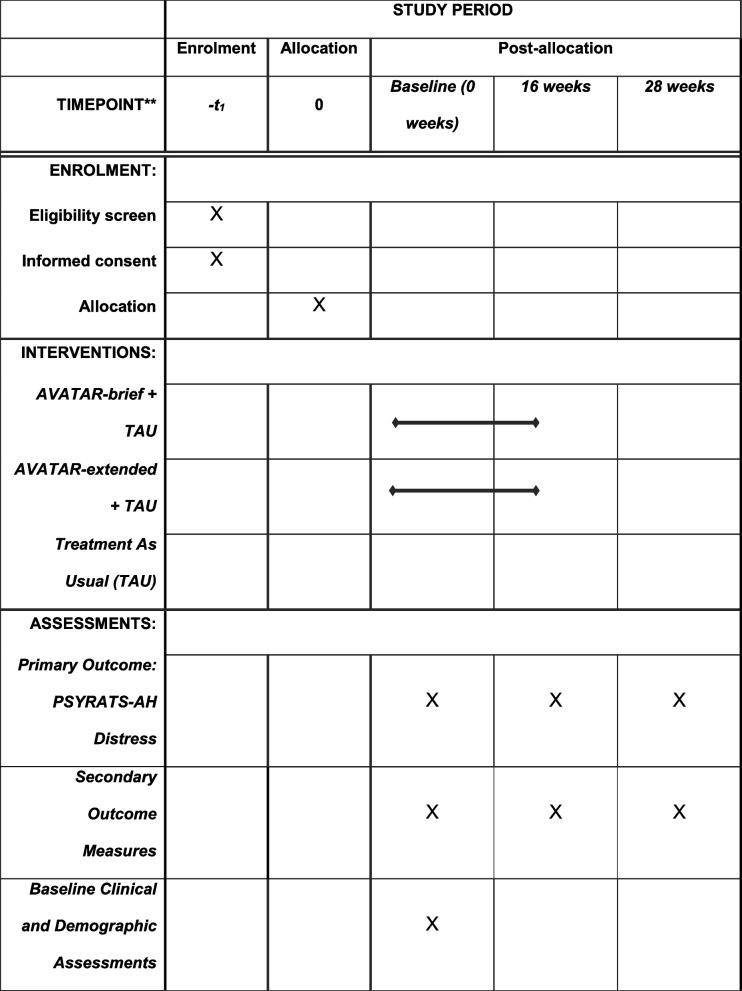
Participant Time Schedule

Figure 1 shows the participant timeline.

### Sample size {14}

We will recruit 345 people in total for the study (approximately 87 per site and 115 per arm). The effect size in the sample size calculation is based on the findings of the previous AVATAR therapy trial [16] which reported clinically meaningful reduction in PSYRATS-AH distress dimension post-treatment of 4.8 points, with an effect size of approximately *d* = 0.8. This has been conservatively reduced for the current trial, to take into consideration the increase in the number of centres, the comparison at both post-treatment and follow-up and a more pragmatic trial design. We are accounting for two formal comparisons (hypothesis 1 and 2).

The study is powered for an overall treatment effect at a 5% alpha level, accounting for 2 multiple comparisons in which the tests are correlated (at *r* = 0.5), giving an alpha level for each test of 0.035. Accordingly, a sample size of 92 per group or 276 in total will have 90% power to detect a minimum clinically significant difference (effect size) of 0.5 standard deviations. We will recruit 345 participants in total at baseline, allowing for an attrition of 20%.

### Recruitment {15}

Participants will be recruited from NHS mental health services linked to each of the four university trial sites (at least 2 NHS settings per site) with identical procedures followed at each. Participants will be identified through close liaison with clinical staff. After clinical staff have confirmed that a potential participant is suitable to be approached (i.e. meets study criteria and no clinical contra-indications), research workers will meet each potential participant to discuss the study, provide written information and time to consider it, respond to questions and seek written informed consent.

We also intend to use recruitment databases or consent for contact initiatives where available to maximise the pool of potential participants. Finally, we can be approached directly by service users interested in taking part and intend to place recruitment posters in the main clinical areas of specialist mental health teams, as well as promoting the trial through online platforms (Twitter, trial website) to facilitate this. In all such instances, we will contact the relevant clinical team and discuss suitability for participation.

## Assignment of interventions: allocation

### Sequence generation {16a}

A randomisation list will be prepared independently by the King’s Clinical Trials Unit using random permuted blocks, stratified by site and baseline voice characterisation An allocation sequence (1:1:1) will be generated, using random permuted blocks of varying size, and stratified by site (4 sites) and baseline voice characterisation (more/less) as defined by meeting the threshold for more highly characterised voice on the characterisation checklist.

### Concealment mechanism {16b}

The allocation sequence will be held independently of the research team and each participant’s allocation revealed via a secure web-based service hosted by King’s Clinical Trials Unit.

### Implementation {16c}

Once a participant has completed baseline assessments, the site trial coordinator will perform the randomisation utilising the web-based service and inform the participant.

## Assignment of interventions: blinding

### Who will be blinded {17a}

Trial coordinators who will not be blind to individual allocations to allow them to inform participants of assignment. The research workers who conduct assessments will remain blinded to the allocation of participants until after the participant has completed their involvement in the trial. These research workers will not be exposed to therapy records whilst they remain blinded. We will be using a system of web-based data entry that ensures assessors do not have access to information in the database that might reveal allocation. Participants will be reminded not to discuss their allocation with research workers when they meet them for the assessment. The senior trial statistician will be blind throughout the study; the trial statistician will be unblind at a group level after the first DMEC meeting. The Statistical Analysis Plan will be prepared before any unblinding of the trial statistician, and only amended by the senior trial statistician.

### Procedure for unblinding if needed {17b}

There are no pre-determined situations in which blinded research workers should become unblinded. Breaks in blindness will be monitored and recorded and where operationally feasible assessments will be allocated to another (blinded) research worker.

## Data collection and management

### Plans for assessment and collection of outcomes {18a}

Assessments will be completed at baseline, after treatment at 16 weeks, and at 28-week follow-up. Participants who have consented will also complete an additional ESM assessment at each time point, responding to questionnaires administered through a smartphone 10 times a day for 6 days. This will be delivered using an application (M-Path, https://m-path.io/landing/) which can be downloaded to the participant’s own phone. Where the participant does not have a smartphone or has concerns about using their own, a smartphone will be provided to them. At the end of the 6 days, the research worker will meet the participant again to complete a debrief questionnaire regarding their experiences of the ESM and to collect the smartphone where this has been provided.

All these assessments will be administered by trained local research workers, who will be supervised by experienced research clinical psychologists. Assessments will be conducted at locations convenient for the participant (at either NHS, University or residential locations).

#### COVID-19

All assessments will take place remotely via a video-conferencing application or telephone, in line with NHS trust and University guidance. Questionnaires should be completed online where possible; that is, where the person has a laptop/tablet and a Wi-Fi connection. Where this is not possible, they will be posted with a stamped addressed envelope to be returned. In the event of social distancing restrictions, additional measures will be taken to manage any distress that occurs with the research team liaising closely with the clinical team caring for the person. The content of the assessment will be as described below.

A sub-sample of participants, approximately 20, who give their consent, will be consecutively recruited across all sites and from both therapy arms in the study to take part in a qualitative interview. This will occur in the final year of the trial, and once those approached have concluded their participation in all assessments in the trial. This interview and the conduct of this sub-study will be developed in collaboration with service user consultant researchers, working with the research team, and will ask participants for feedback on how they found the therapy, taking part in the trial and the technology/software involved.

### Plans to promote participant retention and complete follow-up {18b}

Research workers will maximise engagement with participants and facilitate their completion of follow-up assessments. This includes flexibility around the locations of assessments (home visits where risk assessment is completed) and covering travel expenses for participants. As all outcome measures are through interviews or self-report measures these cannot be completed with participants who discontinue their participation.

### Data management {19}

All data are anonymised at source. No patient identifiable information is recorded on the research assessment records and the computerised database is held centrally and managed by the King’s Clinical Trials Unit. A web-based electronic data capture (EDC) system will be designed, using the InferMed Macro 4 system. The EDC will be created in collaboration with the trial analyst/s and the CI and maintained by the King’s Clinical Trials Unit for the duration of the project. It will be hosted on a dedicated server within KCL. Experience sampling data will be uploaded via a secure network to a Research Electronic Data Capture (REDCap) database managed by the University of Leuven, Belgium who will be conducting the ESM data analysis.

Source data will be entered by recruiting site staff, typically within 7 days of data collection by authorised staff onto the EDC. A full audit trial of data entry and any subsequent changes to entered data will be automatically dated and time-stamped, alongside information about the user making the entry/changes within the system. Database access will be strictly restricted through user-specific passwords to the authorised research team members.

The CI team will undertake appropriate reviews of the entered data, in consultation with the project analyst for the purpose of data cleaning and will request amendments as required. No data will be amended independently of the study site responsible for entering the data*.* In order to ensure the accuracy of the data entered into the database, the primary outcome measure entry will be checked for every participant by comparing the paper record with that on the database. An error rate of no more than 5% is acceptable. This will be completed once all possible assessments for each time point have been completed. If the error rate is higher than 5%, advice will be sought from the trial statisticians regarding further data checking. At the end of the trial, there will be a central process of data cleaning and checking to verify that all the data are complete and correct. At this point, all data will be formally locked for analysis.

Audio recording equipment will be used to record assessments to check fidelity to assessment protocols and to ensure interrater reliability of the PSYRATS-AH (primary outcome) and the Voice Characterisation Checklist (stratification tool). The therapy sessions will be audio recorded (with participant consent) for monitoring the intervention in terms of fidelity and competence. These audio files named with a unique participant identifier will be stored as computer files on secure NHS/ University servers.

All personal data will be kept in a locked filing cabinet in a locked office at the four trial sites and will be accessible only by researchers. Therapy files will be kept in a secure office and are not accessible to the staff collecting the research outcome data. Audio recordings of the therapy will be stored as described above, accessible to the patient's trial therapist and to the senior research clinician supervising that therapist.

### Confidentiality {27}

All data are anonymised at source. A log of contacts with participants including address and other contact details will be kept separate from all the research data. Details necessary to contact participants, and for communication with teams will be stored securely in a locked filing cabinet. A numerical system will be used for computerised information so that individual participants will not be identifiable. Participant consent forms will be retained, kept confidential and stored securely. All identifiable data will be destroyed following a period of 7 years (as determined by relevant information governance policies) after the completion of the trial.

Assessment and therapy sessions will be recorded, with consent, using password-protected smartphone or equivalent devices and data will be transferred to secure central storage as soon as possible. When not in use, devices will be stored in a locked cabinet within a locked office.

### Plans for collection, laboratory evaluation and storage of biological specimens for genetic or molecular analysis in this trial/future use {33}

See above {26b}, there will be no biological specimens collected.

## Statistical methods

### Statistical methods for primary and secondary outcomes {20a}

We will report all participant flow in the study in accordance with the CONSORT principles, with the 2018 extension for reporting social and psychological intervention trials ((CONSORT SPI) [31]). Descriptive statistics will be used to summarise assessments of recruitment, drop-out and completeness of therapy.

The primary outcome of voice-related distress as measured by PSYRATS-AH distress dimension score will be analysed using a mixed effects model including data from all time points. Fixed effects will be site, characterisation, baseline assessment for the outcome under investigation, random allocation, time and time*random allocation interactions. Participant and therapist will be included as random intercepts, with the participants in the TAU arm considered as being in individual clusters of size 1. Marginal treatment effects for each pairwise comparison will be estimated for outcomes at each time point and reported separately as mean adjusted differences in scores between the respective randomised groups with 96.5% confidence intervals and two-sided *p* values (to account for correlated multiple comparisons). For continuous secondary outcomes, the same approach will be followed, and for binary secondary outcomes, logistic mixed models will be used.

The random effect structure will account for repeated measures and clustering due to the partially nested design and allow estimates of separate ICCs in both randomised arms.

Cohen’s d effect sizes at 16 and 28 weeks will be calculated as the adjusted mean difference divided by the sample standard deviation of the outcome at baseline and displayed in a forest plot.

### Interim analyses {21b}

There are no planned interim analyses.

### Methods for additional analyses (e.g. subgroup analyses) {20b}

Causal mediation analysis will be based on parametric regression models [50]. For each mediator separately, this involves estimating a linear model for each mediator with random allocation, baseline outcome, baseline mediator, site and characterisation as covariates, and separately estimating a linear model for each outcome with the mediator, random allocation, baseline outcome, baseline mediator, site and characterisation as covariates. The effect of random allocation on the mediator is multiplied by the effect of mediator on the outcome to estimate the indirect effect, and the effect of random allocation on outcome in the model including mediator is an estimate of the direct effect. The indirect and direct effects sum to the total effect and bootstrapping with 1000 replications will be used to obtain valid standard errors for the causal effects.

For the moderation analyses, these will be conducted by adding interaction terms between random allocation, time and the respective moderators. The difference in treatment effect between unit levels of the moderator can be interpreted as the difference in the estimated treatment effect between a participant with a moderator value at baseline of a+ 1 and a participant with a moderator value at baseline of a.

For analyses involving ESM variables as outcome, an additional level of nesting will be included, with multiple ESM observations (level 1) being nested within time points (level 2) and time points as nested within subjects (level 3).

### Methods in analysis to handle protocol non-adherence and any statistical methods to handle missing data {20c}

The main efficacy analysis will be via intention to treat with data from all participants included in the analysis including those who do not complete therapy. Every effort will be made to follow up all participants for research assessments, and the analysis will use, where appropriate, statistical techniques for handling missing data. All models will be estimated using maximum likelihood estimation, which allows for missing outcome data under the missing at random assumption; we may also use inverse probability weighting to adjust for non-adherence to allocated treatment and other intermediate outcomes as predictors of future loss to follow-up.

### Health economic analyses

The use of services at baseline, 16 weeks and 28 weeks will be measured with the Client Service Receipt Inventory. Service costs will be calculated by combining these data with appropriate unit cost information (including NHS Reference Costs and figures from the University of Kent). These costs will be added to the costs of the therapy (based on staff time, overheads and activity levels). Costs will be compared between the three arms at 16 and 28 weeks using regression models adjusting for baseline differences. (The 28-week comparison will be of the cumulative costs to that time point.) Bootstrap methods will be used to generate confidence intervals around the cost differences. Quality-adjusted life years (QALYs) will be generated using the EQ-5D-5L along with cross-walk method recommended by NICE. We will assume linear change between time points and use area under the curve methods. QALY comparisons will make adjustment for baseline EQ-5D-5L tariffs. Cost-effectiveness will be assessed by generating incremental cost-effectiveness ratios (in the event of non-dominated arms) and uncertainty addressed using cost-effectiveness planes and acceptability curves. Missing cost and EQ-5D-5L data will be imputed using multiple imputation methods on 1000 bootstrapped resamples. We will assume that data are missing at random. The imputation will be conducted on each bootstrapped resample in turn and cost-effectiveness estimates made on each.

#### Plans to give access to the full protocol, participant level-data and statistical code {31c}

The datasets generated and/or analysed during the current study will be available in anonymised form, and the corresponding statistical code, from the research team on reasonable request, subject to review, following the publication of trial results.

## Oversight and monitoring

### Composition of the coordinating centre and trial steering committee {5d}

The trial has been carefully designed to ensure compliance with Good Clinical Practice and scientific integrity. The research programme development, design and implementation will be managed by the Chief Investigator (CI) and the co-applicants, in consultation with service-user consultants and other expert research collaborators from within and outside of the CI's institution. A dedicated Trial coordinator post will assist in the day-to-day management of the project reporting to the (CI). A trial management committee (TMC) will meet monthly, its membership will include the investigators and the Trial coordinator and site coordinators. It will be chaired by the CI and will manage the day–to-day running of the study and ensure good communication between trial sites, receiving monthly reports from each site on recruitment, therapy completion, adverse events, reviewing progress against milestones and finding solutions to problems as they arise. It will oversee the preparation of reports to the Trial Steering Committee (TSC) and Data Monitoring and Ethics Committee (DMEC).

The Clinical Trial Steering Committee (TSC) will meet at least every 6 months. Its purpose is to provide overall supervision of the trial, approving the protocol and amendments, monitoring adherence to the protocol and providing independent advice on all aspects of the trial. A senior clinical academic, independent of the trial team, will be nominated as the chair. The TSC will include senior clinicians, clinical academics and two service users of mental health services with lived experience of psychosis. The principal investigator (the co-applicant lead at each site), co-applicant investigators, trial coordinator and representatives for each clinical site may join the meetings as observers. The Wellcome Trust may appoint up to two representatives who shall have the right to attend TSC meetings in person, by telephone or by other electronic means as observers. Wellcome observers have no right to participate in the decisions of the TSC.

### Patient and Public Involvement (PPI) strategy

Each of the four sites will have a local PPI reference group who will attend regular meetings and be asked to contribute to the delivery of the trial in a range of ways, e.g. reviewing materials, supporting recruitment and dissemination and piloting assessment protocols. These local reference groups will be brought together in annual whole trial PPI meetings with input from expert consultants to ensure effective PPI in AVATAR2. We will also develop and deliver the quantitative interviews with input from these consultants and in collaboration with members of the local PPI reference groups.

#### Composition of the data monitoring committee, its role and reporting structure {21a}

A DMEC will be convened and will meet at least annually and report to the TSC. It will have access to all unblinded trial data and will receive regular reports on adverse events. The DMEC will be notified of any serious adverse events as they occur and will consider whether any interim analyses are warranted, review data (including numbers of participants stratified at each level) and advise the TSC on any ethical or safety reasons why the trial should be prematurely ended. The DMEC chair will be responsible for confirming judgements on the likelihood of any SAE’s relatedness to Clinical Trial involvement as well as the SAE’s intensity and unexpectedness. Membership of the DMEC will be independent of the applicants and of the TSC. An independent senior clinical academic will be appointed as chair and the group will also comprise another independent senior clinical academic and a senior statistician. The CI, trial coordinator and the trial statistician will attend parts of the DMEC meeting to provide reports but will not be members of the DMEC or be present when unblinded data is discussed.

#### Adverse event reporting and harms {22}

The occurrence of adverse events (AEs) will be monitored actively and systematically, following guidance from the Consolidated Standards of Reporting Trials (CONSORT) with the extension for social and psychological interventions, and the extension for reporting of harms. Medical Research Council Guidelines for Good Practice in Clinical Trials will also be followed to ensure good governance of the trial for integrity and participants’ safety and wellbeing.

AEs are defined as any untoward medical occurrence, unintended disease or injury, or untoward clinical signs in service user/patient participants, whether or not related to the therapy or device which require additional support or input from health professionals.

Adverse events will be initially assessed at three levels of intensity: mild, moderate and severe, which reflect the impact of the event on the person at the time. Please note there is a distinction between ‘severe’ and ‘serious’. Seriousness is the criteria for defining regulatory reporting obligations and the following will be considered as serious adverse events (SAE, categories A–C): All deaths (category A), incidents which acutely jeopardise the health or psychological wellbeing of the individual, resulting in immediate hospital admission and/or permanent disability (category B), or resulting in injury requiring immediate medical attention (category C). These SAEs will include but are not limited to (1) hospital admissions; (2) home treatment team involvement; (3) suicide attempts; (4) any violent incident necessitating police involvement (whether victim or accused); (5) self-harming behaviour; and (6) all deaths. In order to ensure active surveillance of harms, at each assessment point, research workers will utilise the interview to actively check for the occurrence of specific AEs. At the completion of the trial, all medical notes will be checked, for the total duration of enrolment, for any previously undisclosed record of AEs. Reasons for withdrawal from the study will also be recorded.

The chair of the DMEC will determine relatedness of the event to the research assessment, therapy or device based on a temporal relationship, and consideration of whether there were insufficient or inadequate instructions for use, deployment, installation, or operation, or any malfunction of the AVATAR software. An adverse event may also be device-related if it was the result of user error or intentional misuse of AVATAR therapy software. The DMEC chair will confirm whether the event is unexpected or unexplained given the participant’s clinical course, previous conditions and history, and concomitant treatments. As detailed in the risk analysis conducted for the CE-marking of AVATAR therapy software, we do not anticipate any serious adverse device effects. We do not anticipate any serious therapy or assessment-related adverse events; this expectation is based on the safety data from the previous AVATAR trial.

All SAEs will be reported immediately to the Chief Investigator and Principal Investigators (for the relevant site) and the independent chair of the Data Monitoring and Ethics Committee (DMEC). At each meeting of the DMEC, or at any time at the request of the DMEC Chair, a full report of AEs will be reviewed.

##### COVID-19

In addition to our recording of adverse events as described above, we will add a category for identification of adverse events as remote-delivery-related. This will ensure we capture any adverse events that may be specifically related to the delivery of assessments or therapy remotely.

#### Frequency and plans for auditing trial conduct {23}

The roles of the TSC and the DMEC are to ensure the trial is conducted to a high standard; these committees are independent from the investigators and sponsor. The DMEC will receive open reports showing summary measures of the data across the sample and will be the only committee to receive closed reports displaying summary measures of the data split by treatment group.

#### Plans for communicating important protocol amendments to relevant parties (e.g. trial participants, ethical committees) {25}

Any important protocol amendments will be submitted for approval to the research ethics committee and the Trial steering Committee for approval, subsequent changes will then be made and recorded in the trial protocol and the ISRTCN registration.

#### Dissemination plans {31a}

It is intended that the results of the study will be reported and disseminated at international conferences and in peer-reviewed scientific journals and will be made available to participants and clinical teams in an accessible format and on the study website. It will also be accessible in print and digital media and presented at stakeholder events. The trial is intended as an important step towards the dissemination of the therapy into routine care, if efficacy is established. If the success of earlier trials is replicated and we can demonstrate that it is feasible to train a larger workforce, next steps would include licensed provision of the AVATAR therapy software and training in therapy to interested parties both in the UK and elsewhere.

## Discussion

AVATAR therapy is a relational therapy for distressing voices involving direct dialogue with a digital representation of the main voice the person hears. Encouraging pilot work [15] led to a fully powered RCT in which AVATAR Therapy was found to be superior to an active control post-therapy with a large effect size of 0.8 [16]. Delivery of AVATAR Therapy in this recent trial resulted in an evolution in our understanding of the core therapy processes and how these might, in principle, differ between a standardised phase 1 focusing on exposure and assertiveness, and a formulation-driven phase 2 incorporating a broader range of treatment targets [14]. Data from the previous trial suggest that anxiety reduction is especially prominent during phase 1, but that other processes during phase 2, including changes in the perceived omnipotence and malevolence of the voice, may contribute to improved treatment outcomes, at least for some participants [17]. The new trial develops these insights and aims to make progress towards increased availability of AVATAR therapy in routine mental health care. In addition to testing efficacy of AVATAR-brief and AVATAR-extended, our examination of mediation will also provide understanding of the mechanisms of therapeutic change. The trial will also provide important information on whether, if the therapy is shown to be effective, this is influenced by the complexity of characterisation of the voice. Other clinical characteristics which might plausibly influence AVATAR treatment effectiveness (e.g. trauma experiences and negative symptoms) will be included in exploratory moderation analyses. In AVATAR2 we will harness the ecological validity of ESM to capture changes in anxiety and the relationship with the voice in daily life at each time-point. Finally, the incremental cost-effectiveness of both AVATAR-brief and AVATAR-extended compared to TAU will also inform future strategies for therapist training and wider provision.

The trial will also allow testing of the AVATAR software platform across multiple sites with participants and therapists recruited from several NHS settings serving both urban and rural populations. It is likely that a proportion of participants will be recruited, assessed and indeed receive therapy remotely. At the time of writing this protocol, the UK is experiencing a second surge in COVID-19 cases. As a result, many clinical services have reduced face-to-face contact for all but urgent purposes and a range of social distancing and other restrictions are periodically imposed in localities or UK nations. Recruitment strategies including meetings with clinicians will be carried out virtually, as required, as will procedures for obtaining consent and data capture. Previously, AVATAR therapy was delivered in clinic settings and although the therapist conducted the dialogue from a separate room, he/she was nevertheless available in person at any point during the therapy. Although remote delivery of therapy is now technically feasible, there are anticipated to be challenges in terms of recruitment and the practical feasibility of delivering sessions increasing the importance of careful monitoring of treatment adherence and fidelity. Whilst we have the technology to deliver therapy via a secure, encrypted system and we can supply the necessary hardware to participants, the extensive geography of the new trial may bring challenges of connectivity and other in-session technical difficulties. The fact that the therapist is not immediately on hand may also be a barrier to recruitment or continued participation especially in the anxiety-provoking early sessions and brief therapy. Communication with the participant’s usual clinical team may also be delayed. Given these potential challenges, detailed standard operating procedures for remote recruitment, assessment and therapy delivery have been developed, the latter informed by recent evidence of successful remote delivery of psychological therapy including trauma-focused work which shares AVATAR therapy’s approach to working in the context of high affect [51]. We anticipate the possibility of conducting sub-analyses that take account of whether data collection and/or therapy were delivered remotely and of the wider covid-19 context.

AVATAR therapy is multifaceted and therapy delivery requires skill, sensitivity, and effective training and supervision [14]. Therapists on the previous trial were four doctoral-level psychologists and one senior consultant psychiatrist, all experienced clinicians with prior expertise in working within a cognitive-behavioural therapy approach for psychosis. The majority of the therapy-specific interventions were developed and refined in the earlier trial. Whilst interest in the approach is growing; for example, with independent pilot work conducted by other research groups [52], the small number of therapists who have delivered AVATAR therapy as pioneered by Julian Leff and developed within our team remains a significant barrier to wider dissemination. The present study extends training to approximately 20 clinicians across at least 8 NHS settings with planned assessments of fidelity and competence in delivering the therapy.

The findings of this trial will provide further data on efficacy and cost-effectiveness across multiple clinical settings, and lead to the optimisation and increased personalisation of the AVATAR therapy approach. This will pave the way, should the findings be positive, to wider therapy training and implementation in the future.

## Trial status

Protocol Version Number: 1.2, Date: 28/10/20.

Recruitment will begin in January 2021 and will be completed in November 2022 with the final follow-up data collected in March 2023.

## Data Availability

The datasets generated and/or analysed during the current study will be available in anonymised form, and the corresponding statistical code, from the research team on reasonable request, subject to review, following the publication of trial results. The Avatar Therapy software will be made available for licence at the end of the trial.

## Abbreviations

AE: Adverse event
AVH: Auditory verbal hallucinations
AVATAR: Audio Visual Assisted Therapy Aid for Refractory auditory hallucinations
BAVQ-R: Beliefs about Voices Questionnaire Revised version
BDI-II: Beck Depression Inventory–II
CAINS: Clinical Assessment Interview for Negative Symptoms
CBTp: Cognitive behavioural therapy for psychosis
CHOICE: The Choice of Outcome in CBT for Psychosis
CI: Chief Investigator
CONSORT: Consolidated Standards of Reporting Trials
CSRI: Clinical Service Receipt Inventory
CTU: Clinical Trials Unit
DASS-21: Depression Anxiety and Stress Scale
DMEC: Data Monitoring and Ethics Committee
EDC: Electronic data capture
ESM: Experience Sampling Methodology
GDPR: General Data Protection Regulation
NHS: National Health Service
ICD: International Classification of Diseases
ISRTCN: International Standard Registered Clinical/soCial sTudy Number
ITQ: International Trauma Questionnaire
KCL: King’s College London
PSYRATS: Psychotic Symptoms Rating Scale
PTSD: Post-traumatic stress disorder
QALY: Quality-adjusted life year
RCT: Randomised controlled trial
REDCap: Research Electronic Data Capture
RQ: Relationships Questionnaire
SAE: Serious adverse event
SAPS: Scale for Assessment of Positive Symptoms
SC: Supportive counselling
SCAN: Schedules for Clinical Assessment in Neuropsychiatry
Mini-TALE: Mini-Trauma and Life Events Checklist
TAU: Treatment as usual
TMC: Trial Management Committee
TVAQ: Trauma Voice Associations Questionnaire
TSC: Trial Steering Committee
VAAS: Voice Acceptance and Action Scale
VPDS: Voice Power Differential Scale
WAI: Working Alliance Inventory
WEMWBS: Warwick Edinburgh Mental Wellbeing Scale
WT: Welcome Trust
